# Supplementary material for: Small molecule regulators of microRNAs identified by high-throughput screen coupled with high-throughput sequencing
Source: Nat Commun. 2023 Nov 21;14:7575. doi: 10.1038/s41467-023-43293-0 (PMC10663445; doi:10.1038/s41467-023-43293-0)
Supplement: Supplementary file 1 — Supplementary Information [file 41467_2023_43293_MOESM1_ESM.pdf]

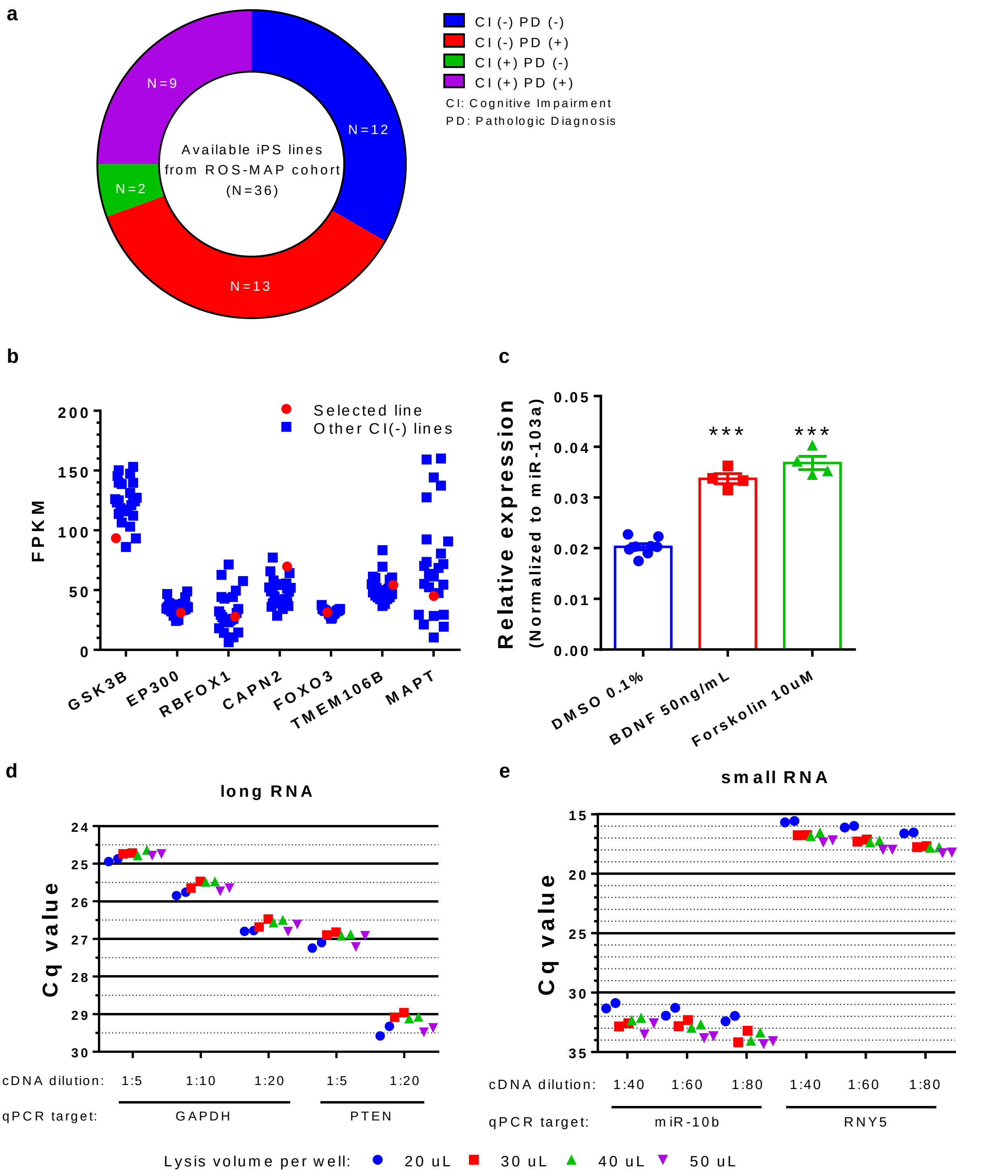

Supplementary Figure S1: Optimization of screen setup. a, ROS-MAP NGN2-iN lines available. **b**, The BR43 line was selected among the NGN2-iNs established from donors without a clinical diagnosis of AD for its median expression of previously validated miR-132 targets. This line came from an 89-year-old female donor without clinical AD diagnosis but with pathological AD diagnosis. **c**, miR-132 was upregulated by positive controls forskolin and BNDF in BR43 NGN-2 iNs (N=8 vs 4 vs 4 biological replicates, unpaired two-tailed Student's t-test). **d-e**, 40 to 50 µL of Takara direct lysis buffer was optimal for direct lysing. 1:10 and 1:60 dilutions were optimal for qPCR on long RNA RT and small RNA RT, respectively (N= 4 technical duplicates). Source data are provided as a Source Data file.

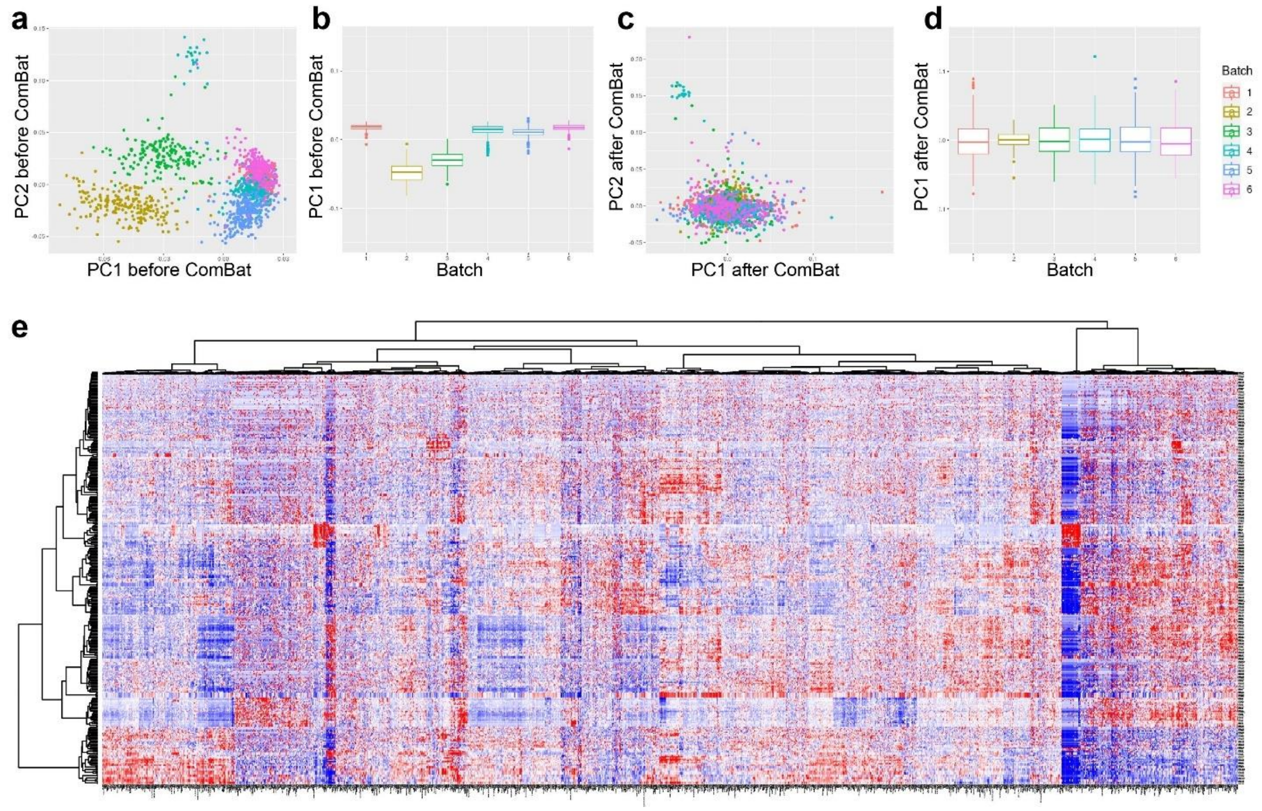

Supplementary Figure S2: Using COMBAT to remove batch effect and enable global analysis. **a**, PCA analysis demonstrates samples' divergence in six batches. **b**, PC1 levels were unequal in the batches. p<0.001, Kruskal-Wallis test. **c**, PCA analysis demonstrates convergence of batches after ComBat adjustment. **d**, PC1 levels became statistically equal after ComBat adjustment. p=0.52, Kruskal-Wallis test. The box plot displays the distribution of the PC1 variable across different batches. The center line of each box represents the median value of PC1 for that batch. The upper and lower hinges of the box represent the third (75th percentile) and first quartiles (25th percentile) of the data, respectively. The whiskers extend from the quartiles to the largest and smallest PC1 values within 1.5 times the interquartile range. Any data points beyond the whiskers are plotted individually and are considered outliers. **e**, Heatmap with hierarchical clustering of all tested compounds (N=1437) against steadily expressed miRNAs (N=338).

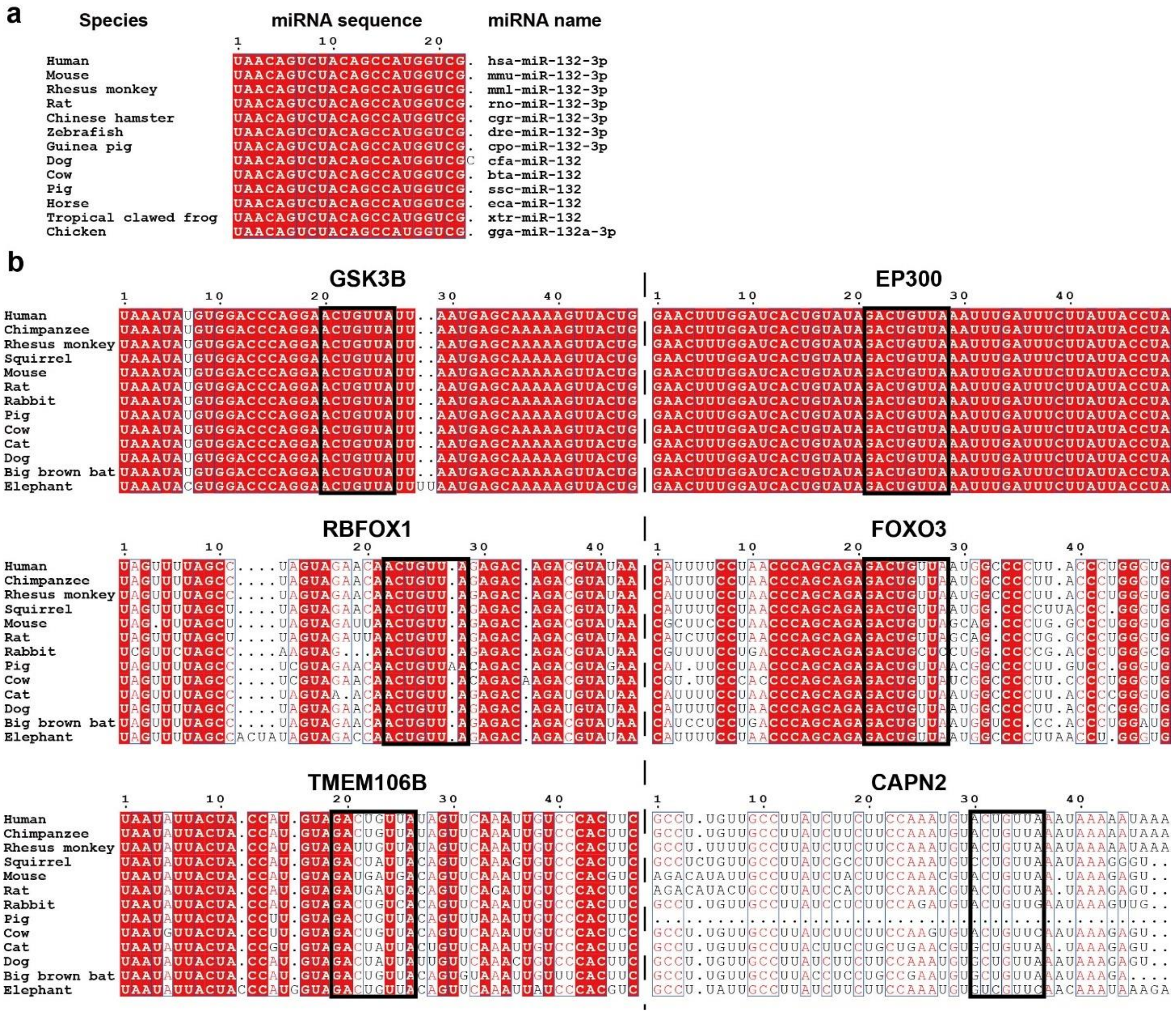

Supplementary Figure S3: Conservation analysis for miR-132 and its targets. a, Conservation analysis for mature miR-132-3p. Sequences of miR-132-3p of multiple species were retrieved from miRBase Release 22.1. **b,** Conservation analysis for targets of miR-132-3p. Binding regions of miR-132-3p to mRNA targets based on TargetScan (Release 8.0), with the best putative binding sites framed by black boxes. Alignment was performed by CLUSTALW v2.1 and visualized by ESPript 3.0.

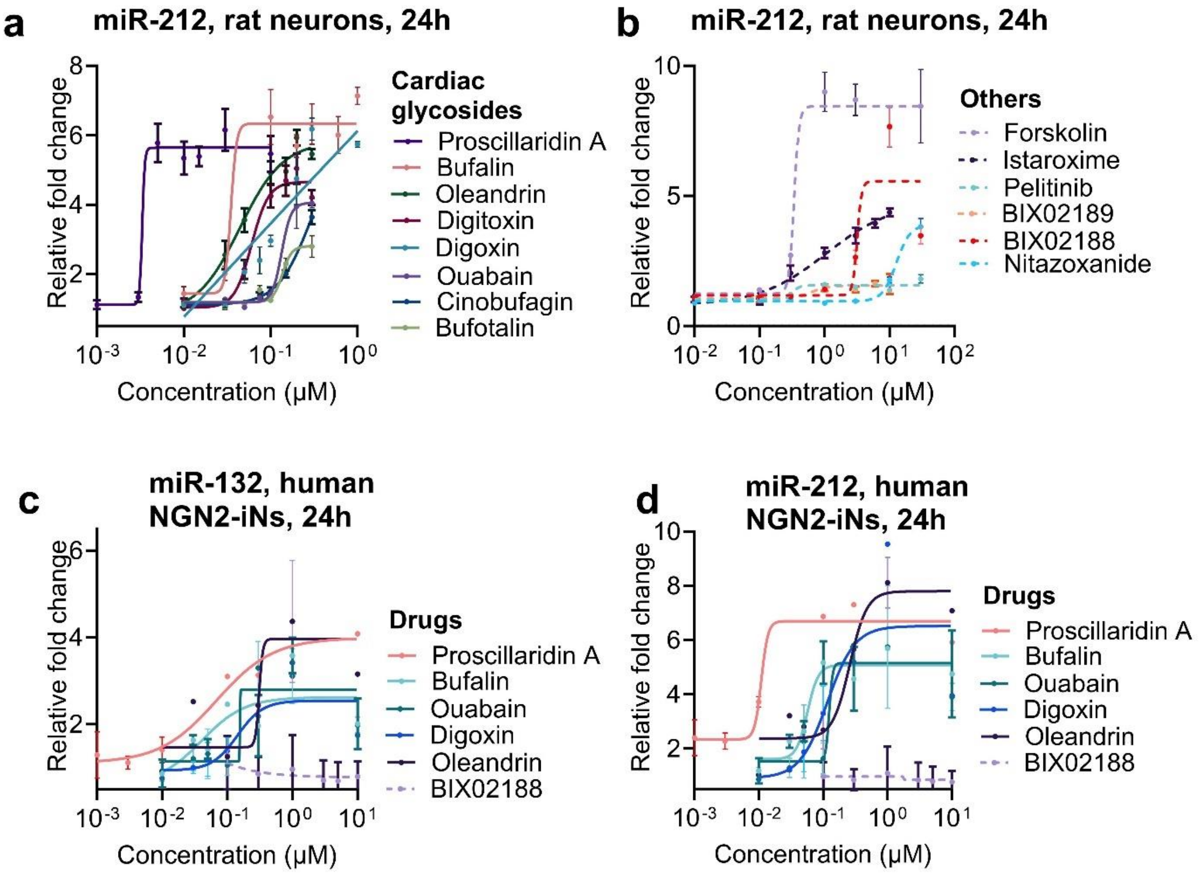

Supplementary Figure S4: Candidate compounds that upregulate miR-132 also upregulated miR-212 in rat primary cortical neurons and human NGN2-iNs. a-b, Dose curve experiments for miR-212 in DIV14 rat neurons after 24h treatment (N=3-6 biological replicates). **c-d**, Dose curve experiments for miR-132 and miR-212 in DIV21 human NGN2-iNs after 24h treatment (N=1-2 biological replicates). Solid lines were used for cardiac glycosides, and dotted lines were used for non-cardiac glycosides. EC_{50} and max fold change were calculated using sigmoidal fit, 4 parameters. Error bars represent mean +/- SD. Source data are provided as a Source Data file.

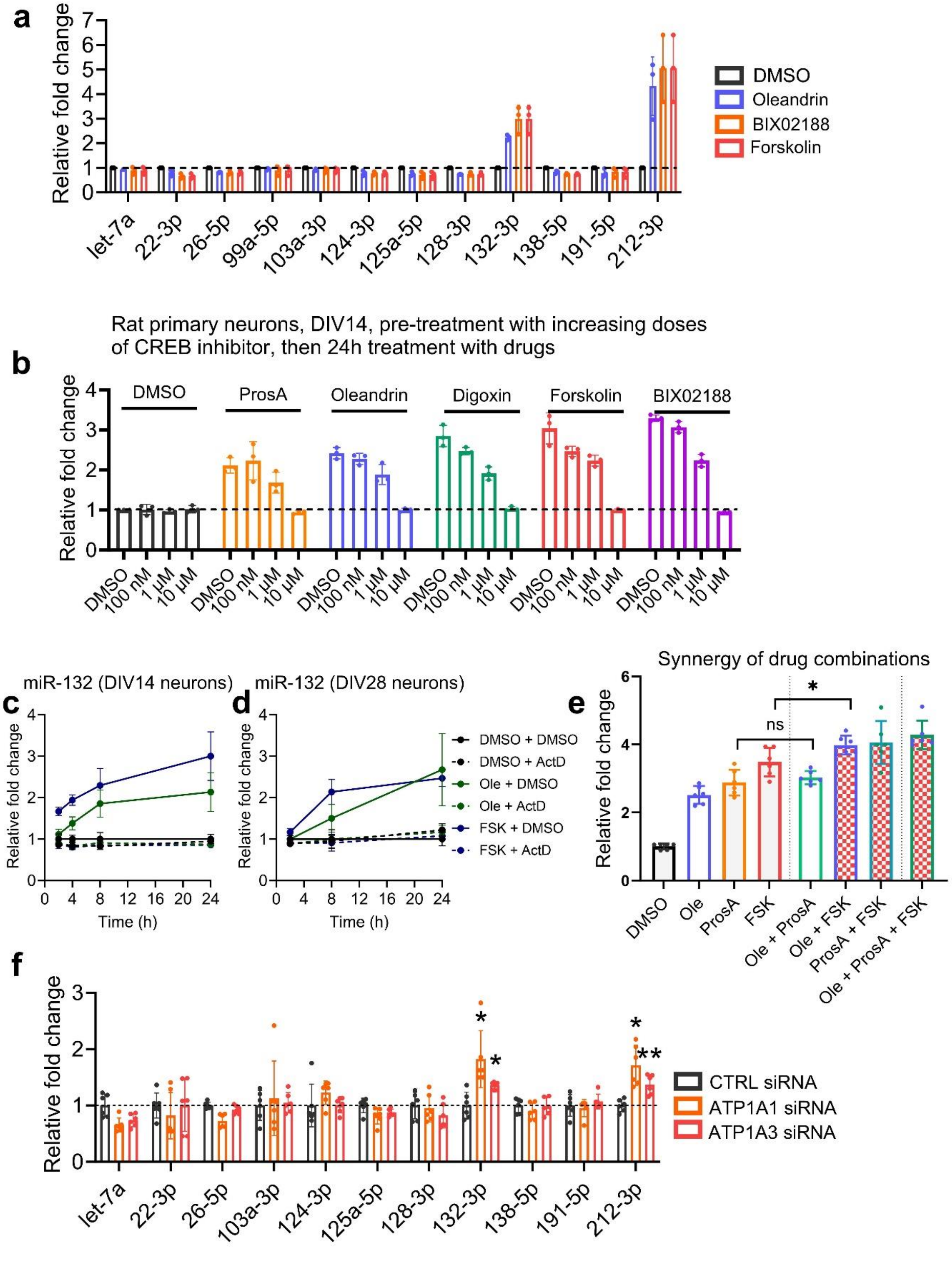

a
Relative fold change
DMSO
Oleandrin
BIX02188
Forskolin
let-7a
22-3p
26-5p
99a-5p
103a-3p
124-3p
125a-5p
128-3p
132-3p
138-5p
191-5p
212-3p
Rat primary neurons, DIV14, pre-treatment with increasing doses of CREB inhibitor, then 24h treatment with drugs
b
Relative fold change
DMSO
ProsA
Oleandrin
Digoxin
Forskolin
BIX02188
DMSO
100 nM
1 μM
10 μM
c
miR-132 (DIV14 neurons)
d
miR-132 (DIV28 neurons)
Relative fold change
Time (h)
DMSO + DMSO
DMSO + ActD
Ole + DMSO
Ole + ActD
FSK + DMSO
FSK + ActD
e
Synergy of drug combinations
Relative fold change
ns
*
DMSO
Ole
ProsA
FSK
Ole + ProsA
Ole + FSK
ProsA + FSK
Ole + ProsA + FSK
f
Relative fold change
CTRL siRNA
ATP1A1 siRNA
ATP1A3 siRNA
let-7a
22-3p
26-5p
103a-3p
124-3p
125a-5p
128-3p
132-3p
138-5p
191-5p
212-3p

Supplementary Figure S5: Cardiac glycosides upregulate miR-132 specifically and transcriptionally in rat primary cortical neurons. **a**, forskolin, oleandrin, and BIX02188 specifically upregulated miR-132/212. Expression was normalized to the geometric mean of all 12 miRNAs tested (N=3 biological replicates). **b**, CREB inhibitor dose-dependently reduced the upregulation of miR-132 by various compounds (N=3 biological replicates). **c-d**, Time-dependent upregulation of miR-132 was completely abolished by pretreatment with 10 μM actinomycin-D before forskolin or oleandrin in DIV14 and DIV28 primary rat neurons (N=4 biological replicates). **e**, Combinations of oleandrin and proscillaridin A did not further synergistically upregulate miR-132, whereas combinations of forskolin and oleandrin did (N=6 biological replicates). **f**, Knocking down ATP1A1 or ATP1A3 upregulated both miR-132 and miR-212 when normalized to the geometric mean of all 11 miRNAs tested (2-way ANOVA, followed by Dunnett's multiple comparisons test comparing to CTRL siRNA, N=6 biological replicates). All error bars show mean +/- SD. Source data are provided as a Source Data file.

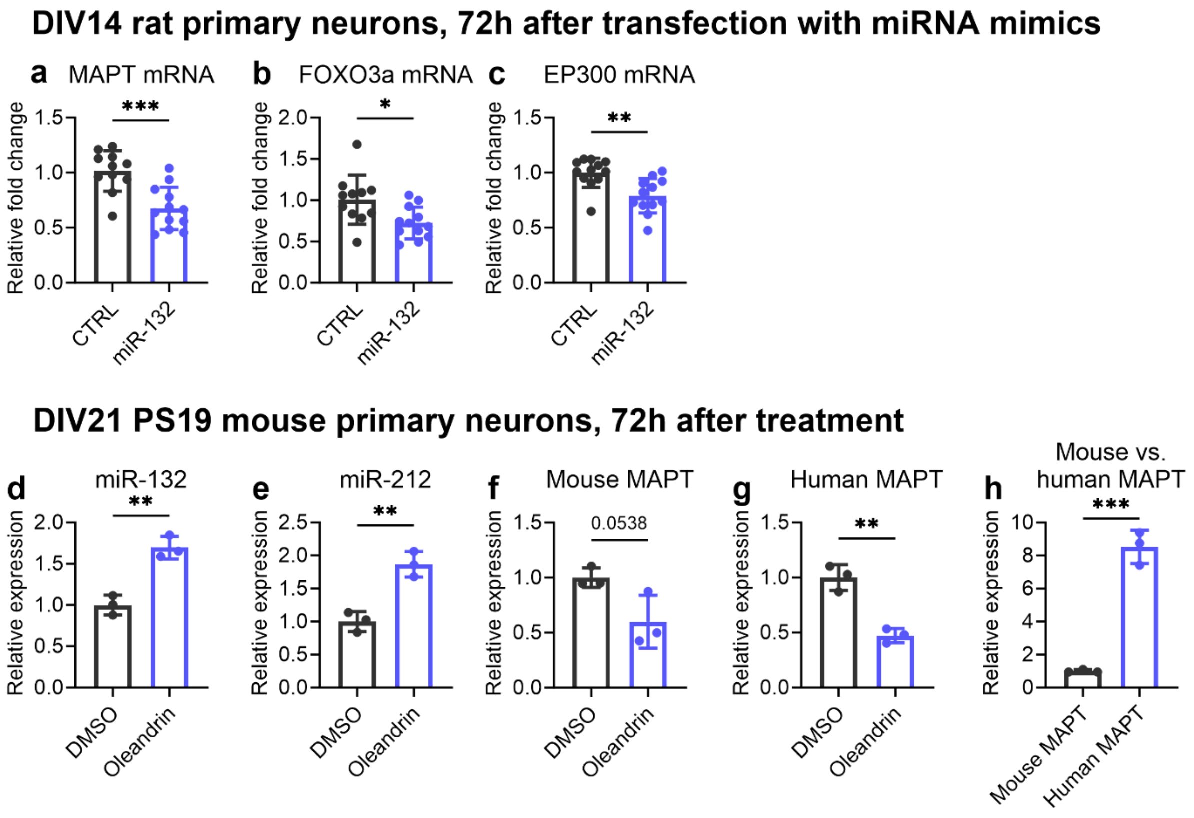

Supplementary Figure S6: Additional effects of miR-132 mimics and cardiac glycosides in rodent neurons. a-c, miR-132 target mRNAs were downregulated 72h after transfection with miR-132 mimics. **a**, MAPT. **b**, FOXO3a. **c**, EP300 (N=11-12 biological replicates, unpaired two-tailed Student's t-test). **d-g**, miR-132 and miR-212 were also upregulated, and mouse and human MAPT mRNA were downregulated by oleandrin in primary PS19 mouse neurons expressing endogenous mouse MAPT and overexpressing human MAPT (N=3 biological replicates, unpaired two-tailed Student's t-test). **h**, human MAPT mRNA was expressed at ~8-fold higher than endogenous mouse MAPT (N=3 biological replicates, unpaired two-tailed Student's t-test). All error bars show mean +/- SD. Source data are provided as a Source Data file.

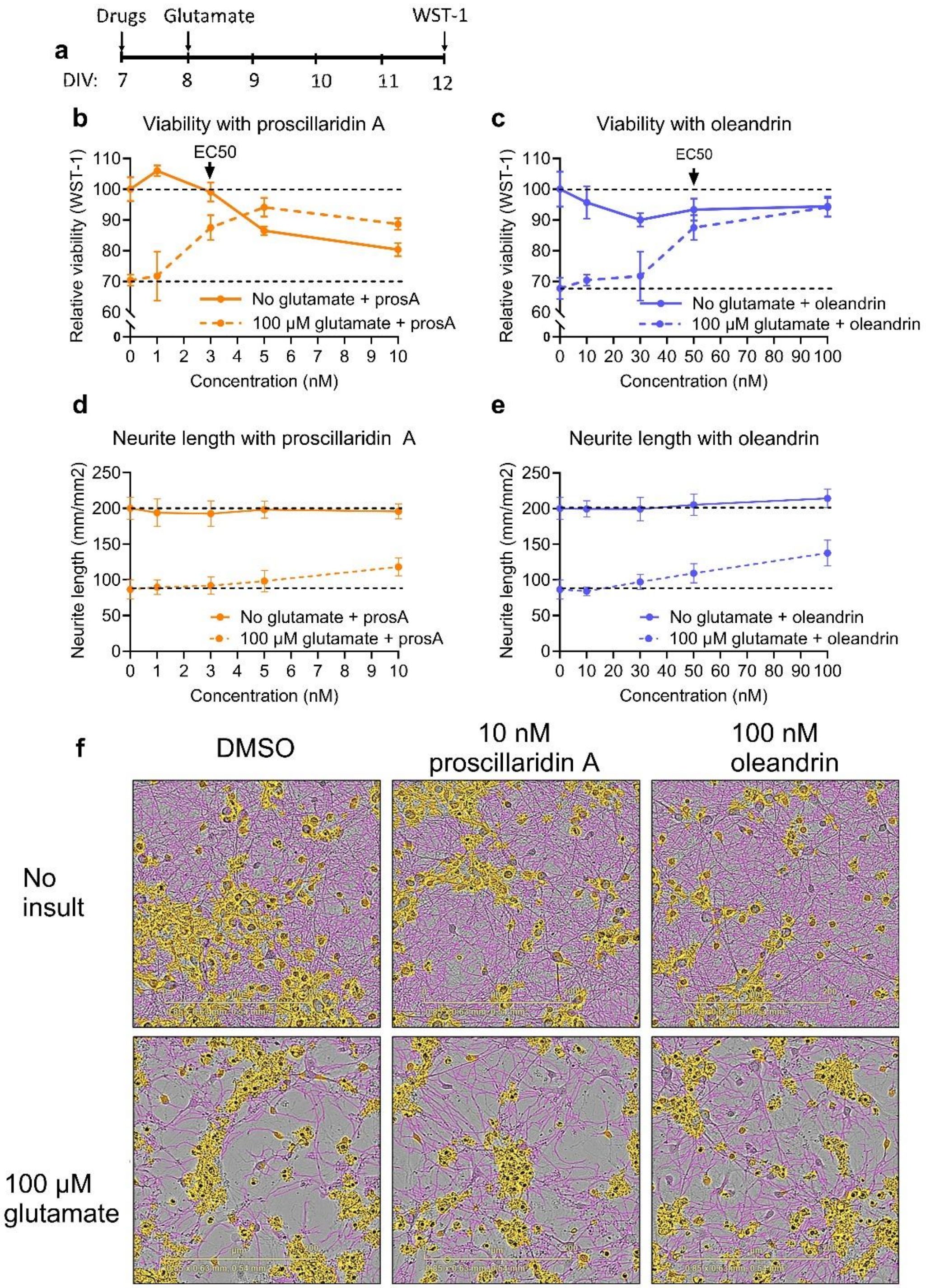

Supplementary Figure S7: Oleandrin and proscillaridin A rescued viability loss from glutamate toxicity but were also mildly toxic in younger in rat primary cortical neurons. a, Experimental scheme. **b**, Proscillaridin A dose-dependently reduced baseline viability (solid line) but also dose-dependently rescued loss of viability due to glutamate (dotted line). **c**, Similar results were obtained for oleandrin. **d-e**, Proscillaridin A and oleandrin did not affect neurite length at baseline and partially rescued loss of neurites due to glutamate. **f**, Representative images of neurons treated with glutamate and proscillaridin A or oleandrin. Cell bodies were highlighted in yellow, and neurites were traced in pink. N=6-8 biological replicates, error bars represent SD. Scale bars are 200 µm. Source data are provided as a Source Data file.

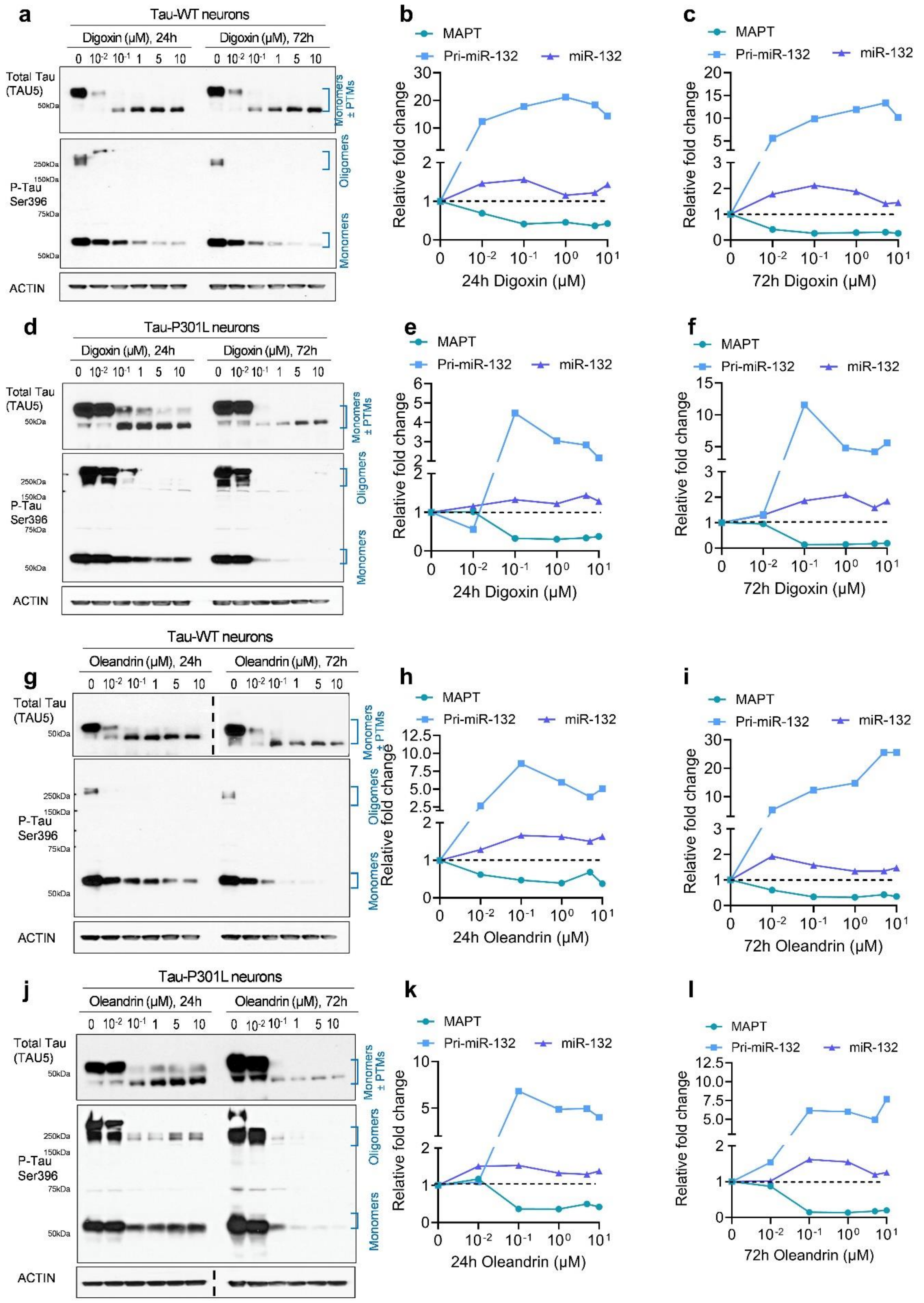
a
Tau-WT neurons
Digoxin (μM), 24h
Digoxin (μM), 72h
0 10-2 10-1 1 5 10
Total Tau (TAU5)
P-Tau Ser396
ACTIN
Monomers ± PTMs
Oligomers
Monomers
b
MAPT
Pri-miR-132
miR-132
Relative fold change
24h Digoxin (μM)
c
72h Digoxin (μM)
d
Tau-P301L neurons
e
f
g
Tau-WT neurons
Oleandrin (μM), 24h
Oleandrin (μM), 72h
h
24h Oleandrin (μM)
i
72h Oleandrin (μM)
j
Tau-P301L neurons
k
l

Supplementary Figure S8: Dose-dependent reduction of Tau in iPSC-neurons treated with cardiac glycosides. Tau WT and P301L neurons were differentiated for 6 weeks, then treated with cardiac glycosides for 24h or 72h. **a**, Representative western blot for WT neurons treated with digoxin. A dose-dependent reduction in total Tau (TAU5) and p-Tau S396 was observed at both 24h and 72h. Similar results were obtained independently 3 times. **b-c**, In parallel, a reduction in MAPT mRNA and an increase in pri-miR-132 RNA were observed (N=1 biological sample). **d-f**, Similar results were also observed in Tau P301L neurons. **g-l,** Similar results were observed for WT and P301L neurons treated with oleandrin. Dotted lines indicated separate Western blots that were put together. Source data are provided as a Source Data file.

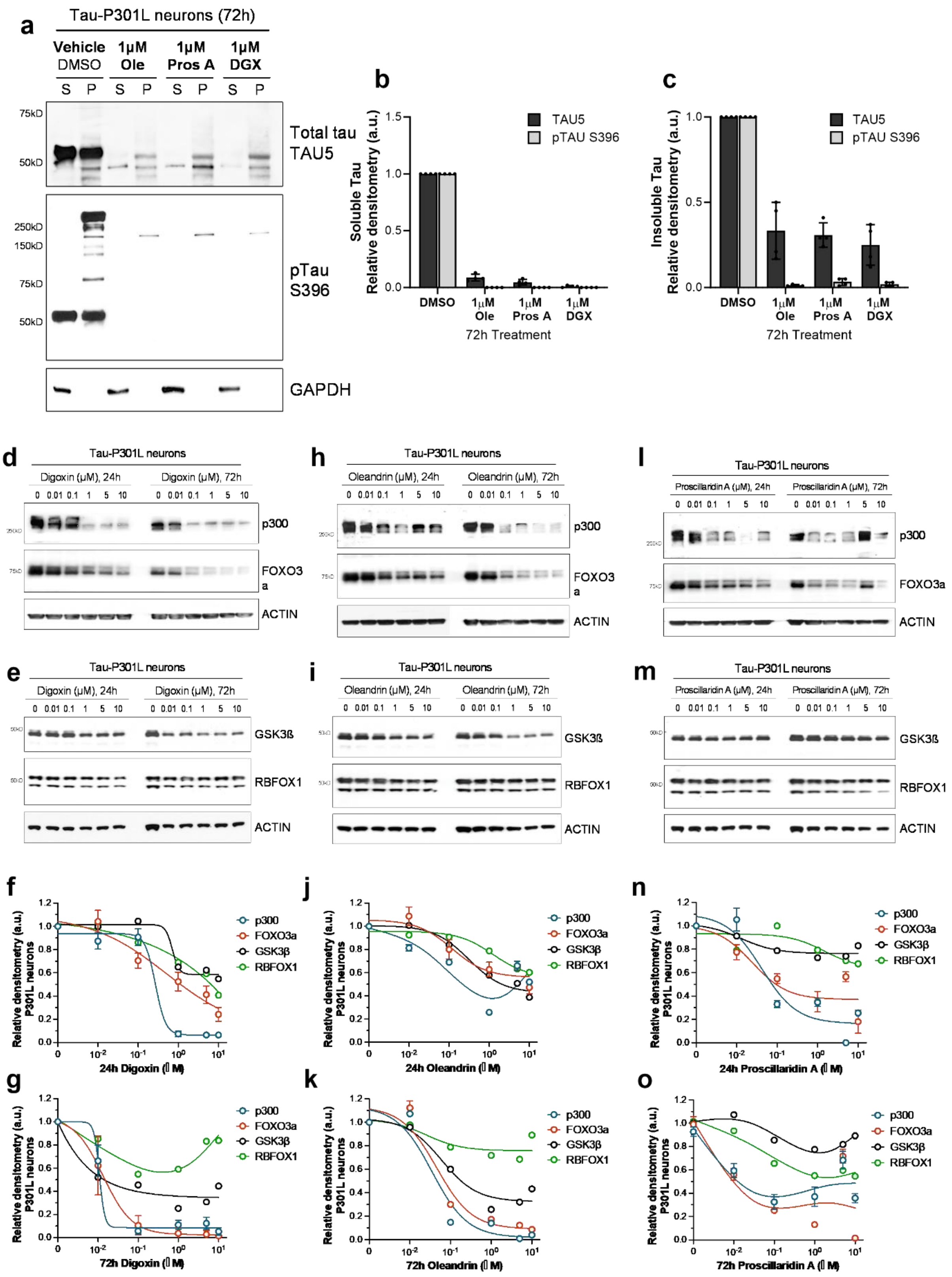
a
Tau-P301L neurons (72h)
Vehicle DMSO
1µM Ole
1µM Pros A
1µM DGX
S P S P S P S P
Total tau TAU5
pTau S396
GAPDH
b
Soluble Tau Relative densitometry (a.u.)
TAU5
pTAU S396
DMSO
1µM Ole
1µM Pros A
1µM DGX
72h Treatment
c
Insoluble Tau Relative densitometry (a.u.)
d
Tau-P301L neurons
Digoxin (µM), 24h
Digoxin (µM), 72h
p300
FOXO3a
ACTIN
h
Oleandrin (µM), 24h
Oleandrin (µM), 72h
l
Proscillaridin A (µM), 24h
Proscillaridin A (µM), 72h
e
GSK3ß
RBFOX1
i
m
f
Relative densitometry (a.u.) P301L neurons
24h Digoxin
j
24h Oleandrin
n
24h Proscillaridin A
g
72h Digoxin
k
72h Oleandrin
o
72h Proscillaridin A
p300
FOXO3a
GSK3β
RBFOX1

Supplementary Figure S9: Cardiac glycosides' effect on Tau solubility and miR-132 targets in P301L iPSC-neurons. iPSC-neurons differentiated for 6 weeks were treated for 72h at concentrations of oleandrin (Ole), proscillaridin A (Pros A) and digoxin (DGX) leading to maximum Tau reduction without detectable toxicity. **a,** Representative western blot analysis of protein lysates generated by detergent fractionation for detection of total Tau (TAU5) and pTau S396 in the soluble (S) and insoluble-pellet (P) fractions. **b-c,** Densitometry analysis of the western blots (N=2). Graph bars represent mean densitometry ± SD for soluble (**b**) and insoluble (**c**) Tau levels relative to vehicle (DMSO). **d-g**, 24h and 72h treatment with digoxin resulted in a dose-dependent reduction in miR-132 targets, including p300, FOXO3a, GSK3β, and RBFOX1 (N=2 biological replicates). **h-o**, Similar results were obtained for oleandrin and proscillaridin A. Error bars represent mean +/- SEM. Source data are provided as a Source Data file.

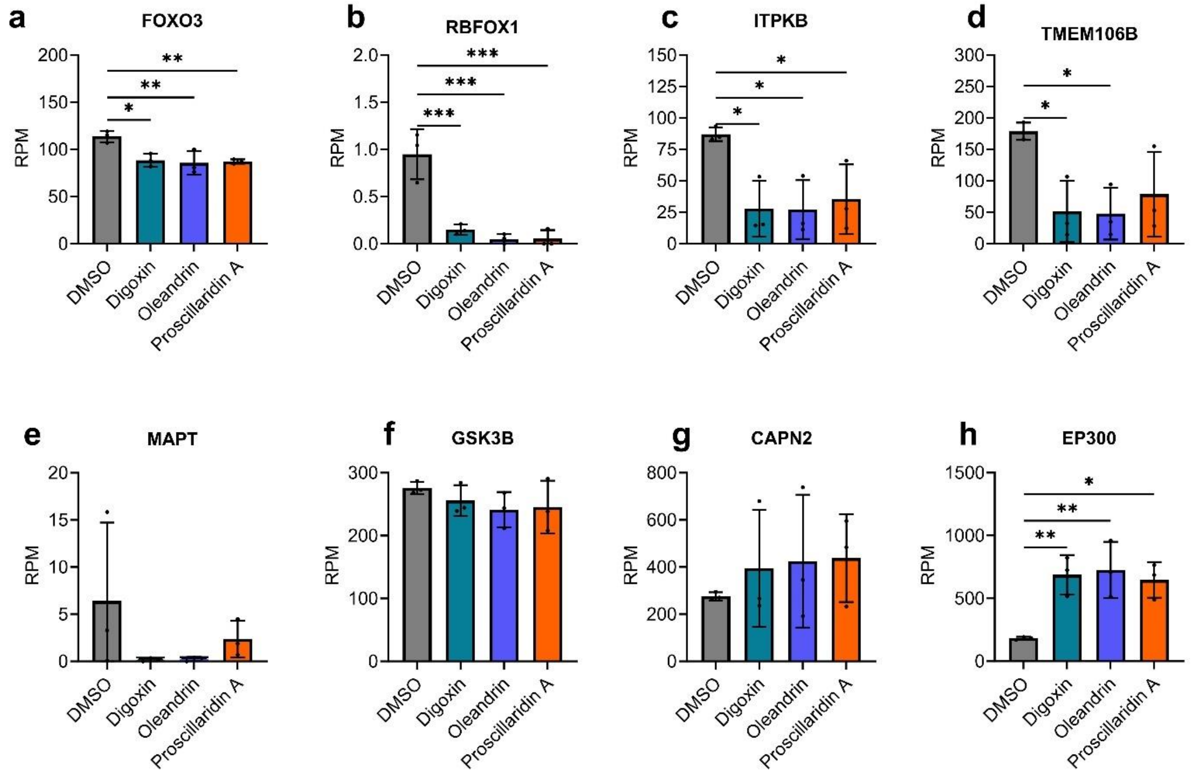

Supplementary Figure S10: RNA-seq RPM results for well-established miR-132 mRNA targets in P301L iPSC-neurons. a-d, FOXO3, RBFOX1, ITPKB, and TMEM106B were significantly downregulated in all or most cardiac glycoside treatment groups. **e,** MAPT was downregulated but was not statistically significant due to the large variation in the control group. **f-g,** GSK4B and CAPN2 were unchanged. **h,** EP300 was upregulated. N= 3, One-way ANOVA, followed by Dunnet’s test for multiple comparisons, error bars show mean +/- S.D. Source data are provided as a Source Data file.

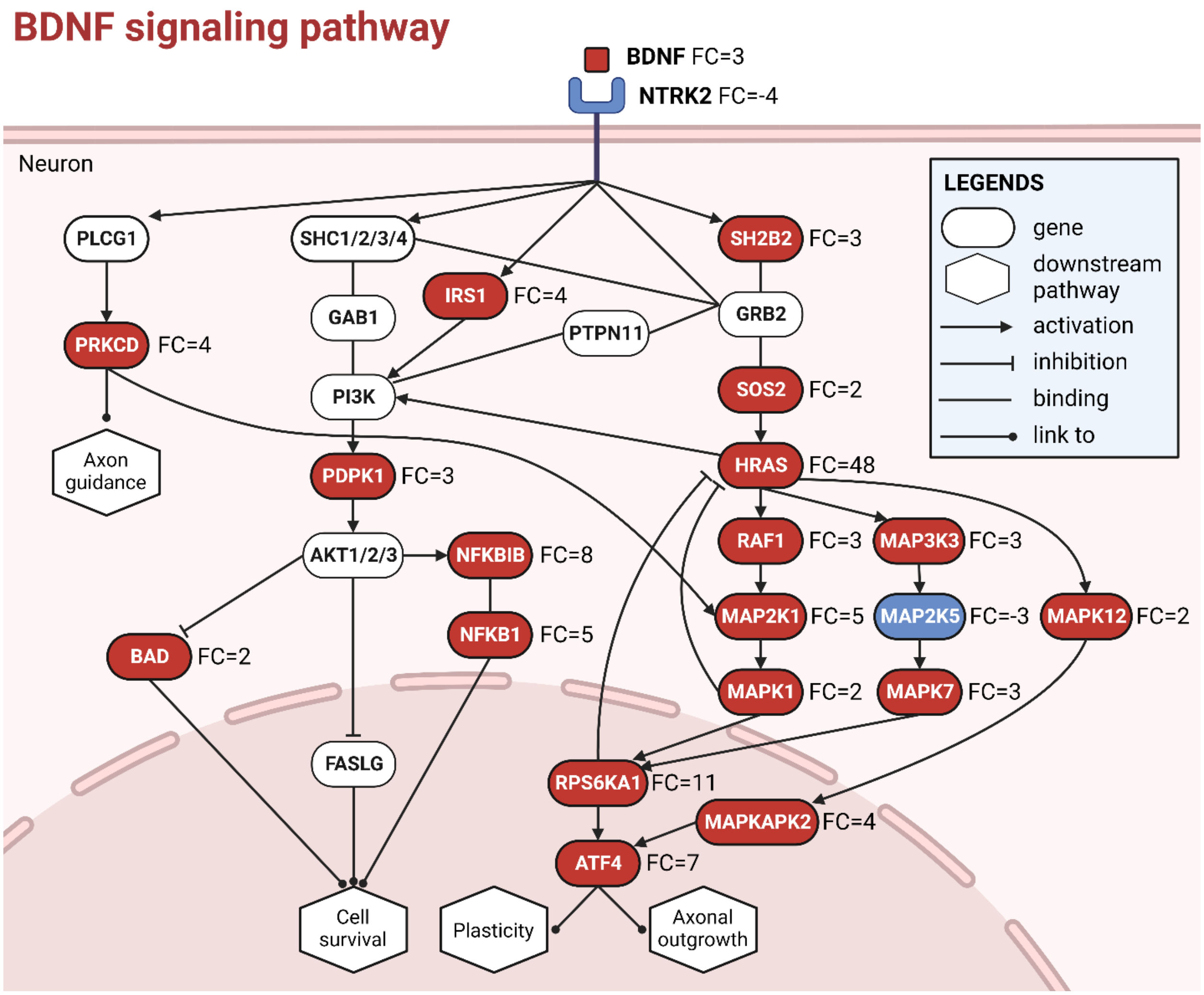

Supplementary Figure S11: BDNF signaling pathway is enriched with genes upregulated by cardiac glycosides in P301L iPSC-neurons. Pathway plot was modified based on KEGG neurotrophin signaling pathway. Genes in red, blue, and white boxes represented upregulated, downregulated, and unaffected ones, respectively. Mean fold changes (FC) among three cardiac glycosides were labeled, of which positive value represented upregulation and vice versa. Created with BioRender.com.
