# Supplementary material for: Small molecule regulators of microRNAs identified by high-throughput screen coupled with high-throughput sequencing
Source: Nat Commun. 2023 Nov 21;14:7575. doi: 10.1038/s41467-023-43293-0 (PMC10663445; doi:10.1038/s41467-023-43293-0)
Supplement: Supplementary file 3 — Description of Additional Supplementary Files [file 41467_2023_43293_MOESM3_ESM.pdf]

Description of Additional Supplementary Files

File Name: Supplementary Data 1
Description: Annotated compounds and count values for all miRNAs

File Name: Supplementary Data 2
Description: Annotated compounds and RPM values for selected miRNAs

File Name: Supplementary Data 3
Description: Annotated compounds and expression level for selected miRNAs after adjusting batch effect with ComBat

File Name: Supplementary Data 4
Description: miRNAs relevant to neurological diseases

File Name: Supplementary Data 5
Description: Validation data for 44 selected compounds

File Name: Supplementary Data 6
Description: EC50 and max fold changes values for selected compounds in primary rat neurons and human NGN2-iNs

File Name: Supplementary Data 7
Description: List of DEGs in human iPSC-neurons after 72h of treatment with digoxin, oleandrin, proscillaridin A and their predicted probablities as miR-132 targets. N= 3 vs 3 for each cardiac glycoside. Cut-off threshold: $|FC|>2$ and adjusted $p<0.05$. Wald test with FDR correction analyzed using DESeq2.

File Name: Supplementary Data 8
Description: Overview of published studies on effects of cardiac glycosides in animal models

File Name: Supplementary Data 9
Description: List of commercial miRNA primers used for miRNA RT-qPCR

File Name: Supplementary Data 10
Description: List of mRNA primer sequences used for mRNA RT-qPCR

File Name: Supplementary Data 11
Description: List of other key resources used in this study
