# Supplementary material for: Small molecule regulators of microRNAs identified by high-throughput screen coupled with high-throughput sequencing
Source: Nat Commun. 2023 Nov 21;14:7575. doi: 10.1038/s41467-023-43293-0 (PMC10663445; doi:10.1038/s41467-023-43293-0)
Supplement: Supplementary file 5 — Reporting Summary [file 41467_2023_43293_MOESM5_ESM.pdf]

nature portfolio

Corresponding author(s): A. Krichevsky, Z. Wei

Last updated by author(s): Oct 20, 2023

Reporting Summary

Nature Portfolio wishes to improve the reproducibility of the work that we publish. This form provides structure for consistency and transparency in reporting. For further information on Nature Portfolio policies, see our Editorial Policies and the Editorial Policy Checklist.

Statistics

For all statistical analyses, confirm that the following items are present in the figure legend, table legend, main text, or Methods section.

n/a	Confirmed	
☐	☒	The exact sample size (*n*) for each experimental group/condition, given as a discrete number and unit of measurement
☐	☒	A statement on whether measurements were taken from distinct samples or whether the same sample was measured repeatedly
☐	☒	The statistical test(s) used AND whether they are one- or two-sided<br>*Only common tests should be described solely by name; describe more complex techniques in the Methods section.*
☒	☐	A description of all covariates tested
☐	☒	A description of any assumptions or corrections, such as tests of normality and adjustment for multiple comparisons
☐	☒	A full description of the statistical parameters including central tendency (e.g. means) or other basic estimates (e.g. regression coefficient) AND variation (e.g. standard deviation) or associated estimates of uncertainty (e.g. confidence intervals)
☐	☒	For null hypothesis testing, the test statistic (e.g. *F*, *t*, *r*) with confidence intervals, effect sizes, degrees of freedom and *P* value noted<br>*Give P values as exact values whenever suitable.*
☒	☐	For Bayesian analysis, information on the choice of priors and Markov chain Monte Carlo settings
☒	☐	For hierarchical and complex designs, identification of the appropriate level for tests and full reporting of outcomes
☒	☐	Estimates of effect sizes (e.g. Cohen's *d*, Pearson's *r*), indicating how they were calculated

Our web collection on statistics for biologists contains articles on many of the points above.

Software and code

Policy information about availability of computer code

Data collection	No customized code was used for data collection
Data analysis	miRNA-seq: Cutadapt, Bowtie, ComBat, UMAP, Silhouette<br>mRNA-seq: Trimmomatic, HiSat2, HTSeq-count using the Galaxy platform, R package DEBrowser with DESeq2.<br>Pathway analysis : Enrichr<br>Promoter binding sites: JASPAR 2022 TFBS via the UCSC genome browser<br>Statistical analyses were performed using Graphpad Prism 9

For manuscripts utilizing custom algorithms or software that are central to the research but not yet described in published literature, software must be made available to editors and reviewers. We strongly encourage code deposition in a community repository (e.g. GitHub). See the Nature Portfolio guidelines for submitting code & software for further information.

Data

Policy information about availability of data

All manuscripts must include a data availability statement. This statement should provide the following information, where applicable:

- Accession codes, unique identifiers, or web links for publicly available datasets
- A description of any restrictions on data availability
- For clinical datasets or third party data, please ensure that the statement adheres to our policy

miRNA-sequencing and mRNA-sequencing data that support the findings of this study were deposited into the Gene Expression Omnibus (GEO) Repository with accession numbers GSE216991 and GSE228348. Source data for Figures 3-6 and Supplementary Figures 1, 4-10 are provided in this paper.

Research involving human participants, their data, or biological material

Policy information about studies with human participants or human data. See also policy information about sex, gender (identity/presentation), and sexual orientation and race, ethnicity and racism.

Reporting on sex and gender	*Use the terms sex (biological attribute) and gender (shaped by social and cultural circumstances) carefully in order to avoid confusing both terms. Indicate if findings apply to only one sex or gender; describe whether sex and gender were considered in study design; whether sex and/or gender was determined based on self-reporting or assigned and methods used. Provide in the source data disaggregated sex and gender data, where this information has been collected, and if consent has been obtained for sharing of individual-level data; provide overall numbers in this Reporting Summary. Please state if this information has not been collected. Report sex- and gender-based analyses where performed, justify reasons for lack of sex- and gender-based analysis.*
Reporting on race, ethnicity, or other socially relevant groupings	*Please specify the socially constructed or socially relevant categorization variable(s) used in your manuscript and explain why they were used. Please note that such variables should not be used as proxies for other socially constructed/relevant variables (for example, race or ethnicity should not be used as a proxy for socioeconomic status). Provide clear definitions of the relevant terms used, how they were provided (by the participants/respondents, the researchers, or third parties), and the method(s) used to classify people into the different categories (e.g. self-report, census or administrative data, social media data, etc.) Please provide details about how you controlled for confounding variables in your analyses.*
Population characteristics	*Describe the covariate-relevant population characteristics of the human research participants (e.g. age, genotypic information, past and current diagnosis and treatment categories). If you filled out the behavioural & social sciences study design questions and have nothing to add here, write "See above."*
Recruitment	*Describe how participants were recruited. Outline any potential self-selection bias or other biases that may be present and how these are likely to impact results.*
Ethics oversight	*Identify the organization(s) that approved the study protocol.*

Note that full information on the approval of the study protocol must also be provided in the manuscript.

Field-specific reporting

Please select the one below that is the best fit for your research. If you are not sure, read the appropriate sections before making your selection.

☒ Life sciences ☐ Behavioural & social sciences ☐ Ecological, evolutionary & environmental sciences

For a reference copy of the document with all sections, see nature.com/documents/nr-reporting-summary-flat.pdf

Life sciences study design

All studies must disclose on these points even when the disclosure is negative.

Sample size	No sample size calculation was performed. Sample sizes were chosen for adequate power based on past experience and the literature. Specific sample sizes were indicated in the figure legends.
Data exclusions	No data were excluded from the analysis.
Replication	All attempts of replication were successful. Each experiment was repeated independently at least three times, except for immunofluorescence staining, which was repeated independently two times.
Randomization	Samples were randomly allocated to treatment conditions.
Blinding	Investigators were blinded to group assignment for data collection and analyses.

Reporting for specific materials, systems and methods

We require information from authors about some types of materials, experimental systems and methods used in many studies. Here, indicate whether each material, system or method listed is relevant to your study. If you are not sure if a list item applies to your research, read the appropriate section before selecting a response.

Materials & experimental systems

n/a	Involved in the study
☐	☒ Antibodies
☐	☒ Eukaryotic cell lines
☒	☐ Palaeontology and archaeology
☐	☒ Animals and other organisms
☒	☐ Clinical data
☒	☐ Dual use research of concern
☒	☐ Plants

Methods

n/a	Involved in the study
☒	☐ ChIP-seq
☒	☐ Flow cytometry
☒	☐ MRI-based neuroimaging

Antibodies

Antibodies used

FOXO3a Abcam ab70315
Tau-5 Invitrogen AHB0042
Tau AT8 Invitrogen MN1020
Tau S396 Abcam ab109390
β-actin Abcam ab3280
Tau S396 Invitrogen 44752G
β-actin Sigma-Aldrich A1978
β-III-Tubulin Sigma-Aldrich T-8660
PSD-95 Neuro-Mab K28/43
SYN1 Synaptic Systems 106-103
Tau K9JA Agilent A002401-2
MAP2 Sigma-Aldrich AB5543
GSK3β Cell Signaling #9315
EP300 Novus Biologicals E15NB100-507SS
FOXO3a Protein Tech/Thermo Fisher 1F12D11
RBFOX1 Thermo Fisher MA5-33104 (A2BP1)
Anti-rabbit IgG Cell Signaling #14708
Anti-mouse IgG Cell Signaling #14709
Anti-rabbit IgG Cell Signaling #7074
Anti-mouse IgG Cell Signaling #7076
Anti-rabbit IgG Alexa Fluor 594 Invitrogen A11012
Anti-mouse IgG Alexa Fluor 488 Invitrogen A11029
Anti-rabbit IgG Alexa Fluor 595 Invitrogen A11032

For Western blot, primary antibodies were diluted 1:1000, secondary antibodies were diluted 1:10,000.
For immunofluorescent staining, Tau K9JA was diluted at 1:1000, MAP2 at 1:1000, Hoechst-33342 at 1:2500. AlexaFluor-conjugated secondary antibodies were diluted at 1:500.

Validation

All antibodies used were commercially available and validated by their respective manufacturers.

Eukaryotic cell lines

Policy information about cell lines and Sex and Gender in Research

Cell line source(s)

Cell line (H. sapiens) BR43 Lagomarsino et al., 2021 (female)
Cell line (H. sapiens) MGH-2046-RC1 Seo et al. 2017 (female)
Cell line (H. sapiens) MGH2069-RC1 Seo et al. 2017 (female)
Rat primary neurons Charles River Prepared from E18 Sprague-Dawley E18 embryos (mixed sex)
Mouse primary neurons Jackson Laboratory Prepared from B6;C3-Tg(Prnp-MAPT*P301S)PS19Vle/J P1-2 pups (mixed sex)

Authentication

iPSC-derived human cell lines were authenticated by short tandem repeat analysis.

Mycoplasma contamination

All cell lines tested negative for mycoplasma.

Commonly misidentified lines
(See ICLAC register)

None

Animals and other research organisms

Policy information about studies involving animals; ARRIVE guidelines recommended for reporting animal research, and Sex and Gender in Research

Laboratory animals	Rat primary neurons were prepared from E18 Sprague-Dawley E18 embryos of mixed sex from timed pregnancy.<br>Mouse primary cortical neuron cultures were prepared from P1 or P2 postnatal pups of mixed sexes from PS19 mouse (B6;C3-Tg(Prnp-MAPT*P301S)PS19Vle/J) breeding pairs.
Wild animals	Study did not involve wild animals.
Reporting on sex	The study was primarily performed on cell models, no animal or human participants were involved. Sex was not considered in the study design and analyses in the reporting summary and method.Sex-based analyses were not performed. Assays were performed on IPSC-derived neurons from female donors, and mixed neurons from male and female rodent pups. The findings do not apply to only one sex or gender.
Field-collected samples	Study did not involve samples collected from the field.
Ethics oversight	This study was carried out in accordance with the recommendations in the U.S. National Institutes of Health Guide for the Care and Use of Laboratory Animals. The protocol was approved by the Institutional Animal Care and Use Committee at Brigham and Women's Hospital. Mice were maintained on a 12:12-h light/dark cycle (7:00 am on/7:00 pm off) with food and water provided ad libitum before experimental procedures

Note that full information on the approval of the study protocol must also be provided in the manuscript.

Plants

Seed stocks	*Report on the source of all seed stocks or other plant material used. If applicable, state the seed stock centre and catalogue number. If plant specimens were collected from the field, describe the collection location, date and sampling procedures.*
Novel plant genotypes	*Describe the methods by which all novel plant genotypes were produced. This includes those generated by transgenic approaches, gene editing, chemical/radiation-based mutagenesis and hybridization. For transgenic lines, describe the transformation method, the number of independent lines analyzed and the generation upon which experiments were performed. For gene-edited lines, describe the editor used, the endogenous sequence targeted for editing, the targeting guide RNA sequence (if applicable) and how the editor was applied.*
Authentication	*Describe any authentication procedures for each seed stock used or novel genotype generated. Describe any experiments used to assess the effect of a mutation and, where applicable, how potential secondary effects (e.g. second site T-DNA insertions, mosiacism, off-target gene editing) were examined.*
